# The Essence of ATP Coupling

**DOI:** 10.5402/2012/827604

**Published:** 2012-05-09

**Authors:** Nikolai Bazhin

**Affiliations:** Institute of Chemical Kinetics and Combustion, Novosibirsk State University, Institutskaya 3, Novosibirsk 630090, Russia

## Abstract

The traditional explanation of ATP coupling is based on the raising of the equilibrium constants of the biochemical reactions. But in the frames of the detailed balance, no coupling occurs under thermodynamic equilibrium. The role of ATP in coupling is not that it provides an increase in the equilibrium constants of thermodynamically unfavorable reactions but that the unfavorable reactions are replaced by other reactions which kinetically are more favorable and give rise to the same products. The coupling with ATP hydrolysis results in the formation of quasistationary intermediate states.

## 1. Introduction

The coupling to ATP hydrolysis is known to be favorable for various biochemical reactions [1]. It is usually explained in terms of equilibrium theory the principle argument of which is a substantial decrease in Gibbs function in the reaction of ATP hydrolysis [1]. No doubt that the latter is a necessary condition with, however, unavailable details of the mechanism. The present work is aimed to demonstrate that the process of coupling occurs in nonequilibrium conditions and that the reaction of ATP hydrolysis gives rise to the products of biochemical reaction of superequilibrium concentrations at the initial stages of the process. Under equilibrium conditions, no coupling is observed despite the presence of ATP.

 It is well known that in real systems, ATP concentration is kept almost constant by means of special synthetic systems. Therefore, it is rather difficult to perceive a detailed mechanism of ATP action in such systems. The present paper considers not only the real systems, but also the thermodynamic and the kinetic behavior of the systems prepared initially in the nonequilibrium state. The processes in these systems occur prior to thermodynamic equilibrium. 

The reaction of ATP hydrolysis, which results in a decrease in a standard value of Gibbs function, is often used as the exergonic reaction, participating in the processes of coupling [1, 2]

(1) $$\begin{array}{c} \text{ATP}=\text{ADP}+\text{Pi},\\ \Delta_{r}G_{1}^{\prime \text{o}}<0, \end{array}$$

where Δ_*r*_ *G*_1_^′o^ = −36.03 kJ/mol [3] for the standard state: pH = 7, *I* = 0.25, and *t* = 25°C. Under these conditions, the equilibrium constant is

(2) $$K_{1}^{\prime }=\frac{{\left[\text{ADP}\right]}_{\text{eq}}\cdot {\left[\text{Pi}\right]}_{\text{eq}}}{{\left[\text{ATP}\right]}_{\text{eq}}}=2\cdot 10^{6}\gg 1.$$

It is assumed (see, e.g., [1, 2]) that if the equilibrium of the reaction (uncoupled system)

(3) $$\begin{array}{c} \text{A}+\text{B}=\text{AB},\\ \Delta_{r}G_{3}^{\prime \text{o}}>0, \end{array}$$

with

(4) $$K_{3}^{\prime }={\frac{{\left[\text{AB}\right]}_{\text{eq}}}{{\left[\text{A}\right]}_{\text{eq}}\cdot \left[\text{B}\right]}}_{\text{eq}}<1$$

is shifted towards reagents, its coupling with ATP hydrolysis provides displacement towards product AB. The summary reaction

(5) $$\begin{array}{c} \text{A}+\text{B}+\text{ATP}=\text{ADP}+\text{AB}+\text{Pi},\\ \Delta_{r}G_{5}^{\prime \text{o}}=\Delta_{r}G_{1}^{\prime \text{o}}+\Delta_{r}G_{3}^{\prime \text{o}}<0 \end{array}$$

is taken as the simplest variant of coupling. In this case, the equilibrium constant of reaction (5)

(6) $$\begin{array}{cc} K_{5}^{\prime } & =\frac{{\left[\text{ADP}\right]}_{\text{eq}}\cdot {\left[\text{Pi}\right]}_{\text{eq}}}{{\left[\text{ATP}\right]}_{\text{eq}}}\cdot {\frac{{\left[\text{AB}\right]}_{\text{eq}}}{{\left[\text{A}\right]}_{\text{eq}}\cdot \left[\text{B}\right]}}_{\text{eq}}\\ & =K_{1}^{\prime }\cdot K_{3}^{\prime }=\exp\left(-\frac{\Delta_{r}G_{5}^{\prime \text{o}}}{RT}\right)>1 \end{array}$$

exceeds unity. It is concluded then [1, 2] that the reaction of ATP hydrolysis provides the energetically unfavorable conversion of reagents A and B into AB.

 However, in the equilibrium system, due to the principle of detailed balance, the concentration of the AB product is in equilibrium with the concentrations of reagents A and B and, thus, is as small as in the absence of ATP. In reaction (3), there is always equilibrium in the equilibrium system independent of other substances. The equilibrium constant of reaction (3) remains unchanged, because its value is determined by the structure of reagents and products and is calculated via a standard change in the Gibbs function. Thus, a thermodynamic coupling of reactions (1) and (3) is impossible in equilibrium conditions. 

 The absence of coupling in equilibrium conditions can be assigned to the absence of the real chemical coupling between reactions (1) and (3) and the independence of both of the reactions. To provide the chemical coupling, intermediate APi (Scheme 1) is usually introduced [4].

**Scheme 1 .**

Consider the following:

(7) $$\begin{array}{c} \text{ATP}\overset{K_{7}}{\to }\text{ADP}+\text{Pi}, \end{array}$$

(8) $$\begin{array}{c} \text{A}+\text{ATP}\underset{K_{8-}}{\overset{K_{8+}}{⇆}}\text{ADP}+\text{Pi}, \end{array}$$

(9) $$\begin{array}{c} \text{APi}+\text{B}\underset{K_{9-}}{\overset{K_{9+}}{⇆}}\text{AB}+\text{Pi}. \end{array}$$

This, however, has no effect on the situation in the equilibrium system. According to the principle of detailed balance, all the feasible processes of the equilibrium system are in equilibrium. Thus, reactions (1) and (3), as well as (8) and (9), are in equilibrium. Due to the presence of the APi product, the amount of the AB product will be slightly less than that in the system with two reactions (1) and (3). It is impossible then to account for coupling in the framework of the equilibrium approach.

Nevertheless, the coupling does exist.

In the real systems, the concentration of the AB product may substantially exceed the equilibrium concentration due to the coupling. What are the reasons for superequilibrium concentrations? One such reason could be the appearance of an additional local minimum of the Gibbs function because of the coupling with ATP hydrolysis. However, an ideal system has only one equilibrium state [5]. (The ideal system is the system in which a chemical potential of each substance is of the form *μ* = *μ* ^o^ + *RT*ln *C*, where *μ* ^o^ is the standard value of the chemical potential, and *C* is the concentration.) Therefore, there is no new equilibrium state which leads to superequilibrium concentrations. Since a thermodynamic system gradually tends to occupy a global minimum, the appearance of superequilibrium concentrations can be assigned to the appearance of quasiequilibrium states that slowly evolve towards the global minimum. We mention the quasiequilibrium state for the following reasons. The matter is that in biochemical systems, most of the chemical reactions proceed in the presence of enzymes. It is assumed then that in their absence, the coupling could be hardly observable despite the fact that enzymes have no effect on the thermodynamic parameters of the system. Enzymes affect only the reaction rate. In this case, this action equally concerns both the direct and inverse reactions. Thus, owing to enzymes, certain reactions are chosen from the variety of feasible reactions. The direct and inverse reactions, accelerated by enzymes, run faster than that of ATP hydrolysis and may be considered quasiequilibrium. During the process, the Gibbs function should decrease regularly despite the appearance of the superequilibrium AB concentrations.

The goal of the present work is to demonstrate that the ATP coupling effect requires fairly fast reactions of the formation of intermediates resulting in the quasistationary state.

## 2. Theoretical Description

 Consider now the kinetic behavior of the system in terms of Scheme 1. In the system, containing ATP, ADP, Pi, A, B, AB, and Pi, only three linearly independent reactions occur, and for convenience, we choose reactions (7), (8), and (9). It is assumed then that the rates of direct and inverse reactions (8) and (9) exceed much the rate of reaction (7). Reactions (8) and (9) proceed in a quasiequilibrium manner. Hence, we get

(10) $$\begin{array}{c} k_{8+}{\left[\text{A}\right]}_{\text{real}}\cdot {\left[\text{ATP}\right]}_{\text{real}}\approx k_{8-}{\left[\text{APi}\right]}_{\text{real}}\cdot {\left[\text{ADP}\right]}_{\text{real}},\\ k_{9+}{\left[\text{APi}\right]}_{\text{real}}\cdot {\left[\text{B}\right]}_{\text{real}}\;\approx k_{9-}{\left[\text{AB}\right]}_{\text{real}}\cdot {\left[\text{Pi}\right]}_{\text{real}}. \end{array}$$

These equations provide expressions for equilibrium constants under quasiequilibrium conditions

(11) $$\begin{array}{c} K_{8,\text{real}}^{\prime }=\frac{k_{8+}}{k_{8-}}=\frac{{\left[\text{APi}\right]}_{\text{real}}\cdot {\left[\text{ADP}\right]}_{\text{real}}}{{\left[\text{A}\right]}_{\text{real}}\cdot {\left[\text{ATP}\right]}_{\text{real}}},\\ K_{9,\text{real}}^{\prime }=\frac{k_{9+}}{k_{9-}}=\frac{{\left[\text{AB}\right]}_{\text{real}}\cdot {\left[\text{Pi}\right]}_{\text{real}}\;}{{\left[\text{APi}\right]}_{\text{real}}\cdot {\left[\text{B}\right]}_{\text{real}}},\\ K_{8,\text{real}}^{\prime }\cdot K_{9,\text{real}}^{\prime }\approx K_{1}^{\prime }\cdot K_{3}^{\prime }. \end{array}$$

In the quasiequilibrium state, we get

(12) $$\begin{array}{cc} K_{1}^{\prime }\cdot K_{3}^{\prime } & \approx K_{8,\text{real}}^{\prime }\cdot K_{9,\text{real}}^{\prime }\\ & =\frac{{\left[\text{ADP}\right]}_{\text{real}}\cdot {\left[\text{Pi}\right]}_{\text{real}}}{{\left[\text{ATP}\right]}_{\text{real}}}\cdot \frac{{\left[\text{AB}\right]}_{\text{real}}}{{\left[\text{A}\right]}_{\text{real}}\cdot {\left[\text{B}\right]}_{\text{real}}}, \end{array}$$

which gives

(13) $$\frac{{\left[\text{AB}\right]}_{\text{real}}}{{\left[\text{A}\right]}_{\text{real}}\cdot {\left[\text{B}\right]}_{\text{real}}}\approx K_{1}^{\prime }\cdot K_{3}^{\prime }\cdot \frac{{\left[\text{ATP}\right]}_{\text{real}}}{{\left[\text{ADP}\right]}_{\text{real}}\cdot {\left[\text{Pi}\right]}_{\text{real}}}.$$

Equation (13) verifies the existence of coupling, because in real conditions [6] [ATP]_real_/([ADP]_real_ · [Pi]_real_) ≥ 1, the value *K*_1_′ · *K*_3_′ > 1, and hence, [AB]_real_/([A]_real_ · [B]_real_) > 1 as compared with the equilibrium ratio [AB]_eq_/([A]_eq_ · [B]_eq_) < 1. Thus, the appearance of quasistationary states results in the formation of intermediates in superequilibrium concentrations. The appearance of the superequilibrium concentrations does not contradict with the thermodynamics as the increasing in Gibbs function due to the superequilibrium concentrations is compensated by decreasing due to reaction of ATP hydrolysis.

Thus, measuring reagent concentrations in the quasistationary conditions, the authors [4] calculated the *K*_1_′ · *K*_3_′ product and, using one of the constants, calculated the second equilibrium constant.

The approximate equation for a change in ATP concentration with time

(14) $$\frac{d\left[\text{ATP}\right]}{dt}\approx -k_{8+}\left[\text{A}\right]\left[\text{ATP}\right]+k_{8-}\left[\text{APi}\right]\left[\text{ADP}\right]$$

shows that at the high A concentration, the time, at which the quasistationary state is reached, is estimated from the formula

(15) $$\tau_{\text{st}}\approx \frac{1}{\left(k_{8+}{\left[\text{A}\right]}_{\text{real}}\right)}.$$

It is worth noting that the *K*_8,real_′ · *K*_9,real_′ product is calculated not only at times close to *τ*_st_, but at longer times as well by realizing the quasistationary conditions.

 Thus, the coupling is reduced to the substitution of reactions (1) and (3) by reactions (7), (8), and (9) which gives rise to the quasiequilibrium state with the formation of the necessary products. This is determined by a favorable change in the Gibbs function upon ATP hydrolysis and the high rates of reactions (8) and (9) as compared with that of ATP hydrolysis. However, in the course of time, the system tends to true equilibrium, at which the concentrations of the products required are very low. This process is also driven by a favorable change in the Gibbs function in the reaction of ATP hydrolysis, as the equilibrium in reaction (7) must take the place.

It is interesting to illustrate the aforementioned experimentally.

## 3. Experimental Examples of Quasiequilibrium State Formation

### 3.1. Acetyl-CoA Formation

 Consider now the production of Acetyl-CoA [4] as an experimental example of reaction kinetics in which the role of ATP is of essence. The reaction of Acetyl-CoA formation from acetate and CoA is impossible due to a small equilibrium constant

(16) $$\begin{array}{c} \text{acetate}+\text{CoA}⟶\text{acetyl-CoA}+{\text{H}}_{2}\text{O},\\ \Delta_{r}G_{16}^{\prime \text{o}}=+33.21\;\text{kJ/mol}. \end{array}$$

However, in the presence of ATP, acetate kinase, and phosphate acetyltrassferase, the reaction

(17) $$\begin{array}{c} \text{acetate}+\text{CoA}+\text{ATP}⟶\text{acetyl-CoA}+\text{ADP}+\text{Pi},\\ \Delta_{r}G_{17}^{\prime \text{o}}=-2.82\;\text{kJ/mol} \end{array}$$

follows the mechanism shown by Scheme 2.

**Scheme 2 .**

Consider the following:

(18) $$\begin{array}{c} \begin{array}{c} \text{ATP}\overset{K_{18}}{\to }\text{ADP}+\text{Pi},\\ \Delta_{r}G_{18}^{\prime \text{o}}=-36.03\;\text{kJ/mol}, \end{array} \end{array}$$

(19) $$\begin{array}{c} \begin{array}{c} \text{ATP}+\text{Acetate}\underset{K_{19-}}{\overset{K_{19+}}{⇆}}\text{APi}+\text{ADP},\\ \Delta_{r}G_{19}^{\prime \text{o}}=+8.61\;\text{kJ/mol}\;K_{9+}, \end{array} \end{array}$$

(20) $$\begin{array}{c} \begin{array}{c} \text{APi}+\text{CoA}\underset{K_{20-}}{\overset{K_{20+}}{⇆}}\text{ACoA}+\text{Pi},\\ \Delta_{r}G_{20}^{\prime \text{o}}=-11.43\;\text{kJ/mol}. \end{array} \end{array}$$

**Notation 1 .**

APi = acetyl phosphate and ACoA = acetyl-CoA. The Δ_*r*_ *G* ^′o^ values were calculated according to [3]. Let us consider results from the second experiment [4, Table  2]. In the paper [4], the data are presented on the concentrations of reaction participants for times 15, 30, and 45 min. The initial concentrations and those at *t* = 1800 s are listed in Table 1.

**Figure 1:**
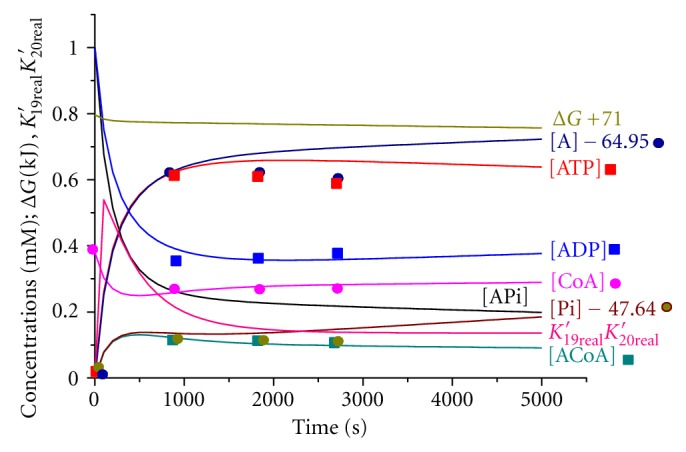
Kinetic curves over small time domain for the second experiment (Table 2 [4]). Experimental data are denoted by the dots.

**Figure 2:**
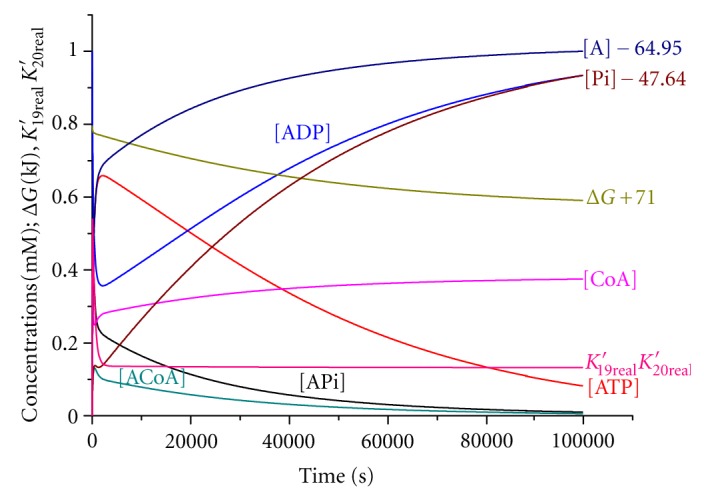
Kinetic curves at long times for the second experiment (Table 2 [4]).

Using the kinetic data on the second experiment [4, Table  2], we have chosen the values of rate constants (Table 2) and the calculated kinetic curves for all reaction participants by the standard Runge-Kutta method as described in the paper [7].  Figure 1 shows the kinetic curves for a short time interval of 0–5000 s, and Figure 2 shows for a longer one of 0–100000 s. The experimental data are well described by theoretical curves.

From the data of Table 1 and Figure 1, the authors [4] concluded that they had reached the equilibrium state of the system. This statement, however, is erroneous for the following reasons. The authors describe the equilibrium state of the system, consisting of seven substances, that is, acetyl-phosphate, ATP, ADP, acetylCoA, CoA, acetate, and phosphate using two linearly independent reactions (19) and (20) of Scheme 2. However, a thermodynamically correct description of this system must include three linearly independent reactions, for example, those present in Scheme 2. The composition of the equilibrium system is easy to calculate from the equations of thermodynamic equilibrium

(21) $$\begin{array}{c} K_{18}^{\prime }=\;\frac{\left(n_{3}^{0}+\xi_{18}+\xi_{19}\right)\cdot \left(n_{7}^{0}+\xi_{18}+\xi_{20}\right)}{n_{2}^{0}-\xi_{18}-\xi_{19}}\;=\;2\cdot 10^{6},\\ K_{19}^{\prime }=\;\frac{\left(n_{1}^{0}+\xi_{19}-\xi_{20}\right)\cdot \left(n_{3}^{0}+\xi_{18}+\xi_{19}\right)}{\left(n_{2}^{0}-\xi_{18}-\xi_{19}\right)\cdot \left(n_{6}^{0}-\xi_{19}\right)}\;=\;0.031,\\ K_{20}^{\prime }=\;\frac{\left(n_{4}^{0}+\xi_{20}\right)\cdot \left(n_{7}^{0}+\xi_{18}+\xi_{20}\right)}{\left(n_{1}^{0}+\xi_{19}-\xi_{20}\right)\cdot \left(n_{5}^{0}-\xi_{20}\right)}\;=\;100.6. \end{array}$$

In these reactions, the equilibrium constants *K*_18_′, *K*_19_′, and *K*_20_′ correspond to reactions (18), (19), and (20) of Scheme 2. For simplicity, we consider the system of volume 1 L. The *n*_*i*_^0^ value describes the number of moles of the *i*-th substance per one liter at the initial stage in moles. The values *ξ*_18_, *ξ*_19_, and *ξ*_20_ describe the chemical extents of reactions in Scheme 2. The chemical extents are given in moles [8] and introduced as follows:

(22) $$\xi =\frac{\left(n_{i}-n_{i}^{0}\right)}{\nu_{i}},$$

where *n*_*i*_ is the amount of the *i*th substance in the system in any stage of the process, and *ν*_*i*_ is a stoichiometric coefficient of the reaction for the *i*th substance. Hence,

(23) $$n_{i}=n_{i}^{0}+\nu_{i}\xi .$$

It is valid for the only reaction in the system. If there are several linearly independent reactions in the system, then

(24) $$n_{i}=n_{i}^{0}+\underset{j}{\sum }\nu_{ij}\xi_{j},$$

where *j* is the number of the reactions, *ν*_*ij*_ is the stoichiometric coefficient for the *i*th substance of the *j*th reaction, and *ξ*_*j*_ is the chemical extent of the *j*th reaction. Usually, in the initial stage of the process, it is convenient to assume that the *ξ*_*j*_ values are zero. The equilibrium constant of the *j*-th reaction is of the form

(25) $$K_{j}=\underset{i}{\prod }C_{j}^{\nu_{ij}}=\underset{i}{\prod }{\left[\frac{\left(n_{i}^{0}+\sum_{j}\nu_{ij}\xi_{j}\right)}{V}\right]}^{\nu_{ij}},$$

where *C*_*j*_ is the equilibrium concentration, and *V* is the system volume. The *ν*_*ij*_ values are positive for products and negative for reagents. When the system volume is 1 L, the equilibrium constant may be given in a simpler form

(26) $$K_{j}={\underset{i}{\prod }\left(n_{i}^{0}+\underset{j}{\sum }\nu_{ij}\xi_{j}\right)}^{\nu_{ij}}.$$

We use chemical extents because the method of chemical extents is a simple and powerful means of describing the equilibrium chemical systems as compared with the method of concentrations. This method is particularly suitable for complex chemical systems, involving several linearly independent reactions in which one and the same substance can serve both the product and the reagent. When equilibrium equations (21) are written using concentrations, they are sure to contain seven unknown values. If we write them in terms of chemical extents, these will involve three unknowns, because the mass conservation laws are taken into account which favors further calculations. When the system volume is 1 L, the number of moles is numerically equal to the concentration, which is also convenient. The scale of changes in chemical extents is determined by the initial amount of reacting substances. The chemical extent may be both positive (reaction is directed to the right) and negative (reaction is directed to the left). It is worth noting that we use the values of chemical extents rather than the *ε* ones, that vary only from 0 to 1. The *ε* values are used but rarely [8].

Equations (21) are solved as follows:

1. the number of phosphate moles in reaction varies but slightly. The range of variations in *ξ*_*j*_ does not exceed 1.01 · 10^−3^ mol. It is assumed then that *n*_7_^0^ + *ξ*_18_ + *ξ*_20_ ≈ *n*_7_^0^. As a result, from equation for *K*_1_′, we find

(27) $$\xi_{18}+\xi_{19}\approx 10^{-3}-24.17\cdot 10^{-12}\;\text{mol},$$

(2) the number of acetate moles in reaction varies but little. Therefore, we assume that *n*_6_^0^ − *ξ*_19_ ≈ *n*_6_^0^. Thus, from equation for *K*_19_′, we find

(28) $$\xi_{19}-\xi_{20}\approx -10^{-3}+48.18\cdot 10^{-12}\;\text{mol},$$

(3) in equation for *K*_20_′, we assume that *n*_7_^0^ varies but slightly and substitute the value for *ξ*_19_ − *ξ*_20_ to determine *ξ*_20_

(29) $$\xi_{20}\approx 38.48\cdot 10^{-12}\;\text{mol}.$$

Hence,

(30) $$\begin{array}{c} \xi_{18}\approx 1.01\cdot 10^{-3}-110.83\cdot 10^{-12}\;\text{mol},\\ \xi_{19}\approx -10^{-3}+86.66\cdot 10^{-12}\;\text{mol},\\ \xi_{20}\approx 38.48\cdot 10^{-12}\;\text{mol}. \end{array}$$

The equilibrium constants in (21) are given to within 2% using the values *ξ*_18_, *ξ*_19_, and *ξ*_20_. The values of equilibrium concentrations are listed in Table 1 which shows substantial difference in the values of equilibrium concentrations and those in the quasiequilibrium state. 

Figures 1 and 2 demonstrate the curves for the change in the Gibbs function (Δ*G* + 71) kJ of the reacting system with time. The value of the Gibbs function is observed to decrease monotonically with time.

The time dependence of the Gibbs function was calculated for a solution of volume 1 L from the expression

(31) $$G\left(t\right)=\underset{i}{\sum }n_{i}\left(t\right)\left[\mu_{i}^{\text{o}}+\;RT\;\ln C_{i}\left(t\right)\right].$$

The standard *μ*_*i*_^o^ values were taken from [3], where *n*_*i*_(*t*) is the amount of the *i*th reagent in the system of volume 1 L at time *t*, and *C*_*i*_(*t*) is the concentration of the *i-*th reagent at time *t*.

As follows from Figure 1, the quasistationary regime is realized at times exceeding 1000 s, which is in accord with the calculations performed by (15). The reaction rates at 1800 s are summarized in Table 2. The rates of direct and inverse reactions (19) and (20) are observed to be close to each other and exceed the ATP hydrolysis rate by about order of magnitude. Thus, the quasistationary regime is satisfied. Figures 1 and 2 show that the product of the equilibrium constants of reactions (19) and (20) is held constant within a time domain of 3000–100000 s.

As follows from Figure 2, in the course of time, the system tends to true equilibrium at which the concentration of the ACoA product is very low, and there is no point in discussing coupling in the case of thermodynamic equilibrium. Thus, the experiment is in fair agreement with the theoretical concepts.

It is readily seen that in the ATP coupling of essence are not only thermodynamic factors but also the kinetic ones. For example, let us decrease the rate constants of reactions (20), *k*_20+_ and *k*_20−_ by a factor of 10. The equilibrium constant and the Δ_*r*_ *G*_20_^′o^ values remain unchanged. However, the maximum amount of ACoA and the time during which a maximum is reached vary substantially (Table 3). Thus, ATP is sure to produce desired products under quasistationary conditions without varying the equilibrium constant of unfavorable reactions.

### 3.2. Phosphorylation of Glucose

 Consider now the process of glucose phosphorylation. A change in the Gibbs function in the direct process of phosphorylation using phosphate amounts to 11.57 kJ/mol. It is assumed then that the equilibrium constant of glucose phosphorylation increases by 2 · 10^5^ due to ATP hydrolysis [6]. However, the equilibrium system, consisting of ATP, ADP, Pi, glucose, and glucose-6-phosphate, can be described in terms of two linearly independent reactions, either uncoupled

(32) $$\begin{array}{c} \begin{array}{c} \text{ATP}=\text{ADP}+\text{Pi},\\ \Delta_{r}G_{32}^{\prime \text{o}}=-36.03\;\text{kJ}/\text{mol},\\ K_{32}^{\prime }=2.0\cdot 10^{6}, \end{array} \end{array}$$

(33) $$\begin{array}{c} \begin{array}{c} \text{glucose}+\text{Pi}=\text{glucose-6-phosphate},\\ \Delta_{r}G_{33}^{\prime \text{o}}=+11.57\;\text{kJ}/\text{mol},\;\\ K_{33}^{\prime }=9.4\cdot 10^{-3} \end{array} \end{array}$$

or coupled

(34) $$\begin{array}{c} \begin{array}{c} \text{ATP}=\text{ADP}+\text{Pi},\\ K_{34}^{\prime }=K_{32}^{\prime }, \end{array} \end{array}$$

(35) $$\begin{array}{c} \begin{array}{c} \text{glucose}+\text{ATP}=\text{glucose-6-phosphate}+\text{ADP},\\ \Delta_{r}G_{35}^{\prime \text{o}}=-24.41\;\text{kJ}/\text{mol},\\ K_{35}^{\prime }=1.9\cdot 10^{4}. \end{array} \end{array}$$

It is worth noting that the equilibrium state is independent of the choice of linearly independent reactions. As follows from (32) and (33) or (34) and (35), in the equilibrium state, the system mainly contains ADP, Pi, glucose, and the small amounts of ATP and glucose-6-phosphate. Indeed, let us consider the system with initial concentrations:

(36) $$\begin{array}{c} \left[\text{ATP}\right]=a_{0}=10^{-3}\;\text{M},\\ \left[\text{glucose}\right]=g_{0}=10^{-3}\;\text{M},\\ \left[\text{ADP}\right]\left[\text{Pi}\right]=\left[\text{glucose-6-phosphate}\right]=0\;\text{M}. \end{array}$$

The equilibrium constants are

(37) $$\begin{array}{c} K_{32}^{\prime }=\xi_{32}\cdot \frac{\left(\xi_{32}-\xi_{33}\right)}{\left(a_{0}-\xi_{32}\right)},\\ K_{33}^{\prime }=\frac{\xi_{33}}{\left[\left(\xi_{32}-\xi_{33}\right)\cdot \left(g_{0}-\xi_{33}\right)\right]},\\ K_{34}^{\prime }=\frac{\xi_{34}\cdot \left(\xi_{34}+\xi_{35}\right)}{\left(a_{0}-\xi_{34}-\xi_{35}\right)},\\ K_{35}^{\prime }=\frac{\xi_{35}\cdot \left(\xi_{34}+\xi_{35}\right)}{\left[\left(a_{0}-\xi_{34}-\xi_{35}\right)\cdot \left(g_{0}-\xi_{35}\right)\right]}, \end{array}$$

where *ξ*_32_, *ξ*_33_, *ξ*_34_, and *ξ*_35_ are the extents of reactions (32), (33), (34), and (35), accordingly, at equilibrium. The extents of the reactions at equilibrium

(38) $$\begin{array}{c} \xi_{32}=\left(10^{-3}-\;0.5\cdot 10^{-12}\right)\;\text{mol},\\ \xi_{33}=9.4\cdot 10^{-9}\;\text{mol},\\ \xi_{34}=\left(10^{-3}-9.4\cdot 10^{-9}-0.5\cdot 10^{-12}\right)\;\text{mol},\\ \xi_{35}=9.4\cdot 10^{-9}\;\text{mol} \end{array}$$

satisfy (37). In this case, the chemical extents were calculated in the same manner as for the system of (21).

The extents of reactions (33) and (35) of glucose-6-phosphate formation in the equilibrium conditions are very small and equal to each other. Thus, no coupling is observed in the equilibrium system. The coupling manifests itself at fairly short times due to reaction (35). As the rate constants of the direct and inverse reactions are relatively high, the quasistationary state arises which gives a quantity of glucose-6-phosphate. Further, the quasistationary equilibrium in (35) shifts gradually to the left due to the ATP hydrolysis in reaction (1) which is unobservable for real biochemical systems, because glucose-6-phosphate enters rather quickly into other reactions, and the ATP concentration is kept constant. In real biochemical systems, the ATP concentration is much higher than the equilibrium one, which favors the fast reactions of phosphorylation.

## 4. Conclusions

ATP is an effective phosphorylating agent which, due to favorable change in Gibbs function and in the presence of suitable enzymes, provides fast phosphorylation resulting in superequilibrium concentrations of phosphorylation products in quasistationary states. A quasistationary system is a point at the Gibbs function surface which slowly tends to a global minimum in the course of ATP hydrolysis. The closer to the global minimum, the lower the concentration of the phosphorylation products. In real biochemical systems, the quasistationary phenomena are unobservable, because the ATP concentration is kept almost constant at the level which substantially exceeds the equilibrium one which provides (with the help of enzymes) the fast processes of phosphorylation. In equilibrium systems, no coupling is observed. This coupling phenomenon is attained by combining thermodynamic and kinetic factors.

Problems of using ATP as energy carrier in biochemical systems have been discussed in the Appendix.

## Figures and Tables

**Table 1:** Initial concentrations, concentrations at *t* = 1800 s, and equilibrium concentrations, mM for the second experiment of Table 2 [4].

*N*	1	2	3	4	5	6	7
Substance	Acetyl-phosphate	ATP	ADP	AcetylCoA	CoA	Acetate	Phosphate
Initial concentrations	1.0	0.01	1.0	0	0.38	64.95	47.86
Concentrations at *t* = 1800 s	0.22 (calculated)	0.607	0.362	0.110	0.268	65.56	47.97
Equilibrium concentrations at *t* → *∞*, calculated this work	48.8 · 10^−9^	24.17 · 10^−9^	1.01	38.48 · 10^−9^	0.38	65.95	48.87

**Table 2:** Reaction rate constants and reaction rates at 1800 s.

Constant	*k*_18_ s^−1^	*k*_19+_, (mM·s)^−1^	*k*_19−_, (mM·s)^−1^	*k*_20+_, (mM·s)^−1^	*k*_20−_, (mM·s)^−1^
Constant value	0.00003	0.000006	0.0035	0.003	0.00004

Reactions rate at *t* = 1800 s, mM/s	1.6 · 10^−5^	2.3 · 10^−4^	2.8 · 10^−4^	1.8 · 10^−4^	2.0 · 10^−4^

**Table 3:** The effect of rate constant on the maximum amount of ACoA.

Rate constants of reaction (20)	*k*_20+_, (mM·s)^−1^	*k*_20−_, (mM·s)^−1^	The time at which the maximum ACoA concentration is reached, s	[ACoA]_max_, mM
Rate constants are the same	0.003	0.00004	600	0.13
Rate constants are decreased 10 times	0.0003	0.000004	7700	0.082

**Table 4:** The change in Gibbs function in reactions with and without ATP.

No	Reaction without ATP	Δ_r_ *G* ^′o^ without ATP, kJ/mol	Δ_r_ *G* ^′o^ with ATP, kJ/mol
(1)	Glucose + Pi = glucose6phos + H_2_O	+11.62	−24.42
(2)	Fructose6phos + Pi = Fructose16phos + H_2_O	+12.79	−23.25
(3)	Pyruvate + CO_2,total_ = oxaloacetate + H_2_O	+27.22	−8.81
(4)	2NH_3_ + CO_2,total_ = urea + 2H_2_O	+30.18	−5.85
(5)	acetate + CoA = acetylCoA +H_2_O	+33.21	−2.82
(6)	Citrate + CoA = acetylCoA + oxaloacetate + H_2_O	+36.61	+0.58

## Appendix

The use of ATP, resulting in a −36.03 kJ/mol change in Gibbs function upon hydrolysis, seems rather effective, as it provides coupling for very many reactions some of which are summarized in Table 4.

If the Gibbs function, Δ_*r*_ *G* ^′o^, changed upon ATP hydrolysis by another value, for example, by −26 kJ/mol, the latter four reactions, presented in Table 4, either would stop or their rate would substantially slow down. Thus, the application of ATP with Δ_*r*_ *G*_1_^′o^ = −36,03 for coupling with other reactions appears to be valid.

In this case, however, there is another more intricate problem. The ADP molecule also exhibits similar changes in both Δ_*r*_ *G* ^′o^ and Δ_*r*_ *H* ^′o^. Thus, the question arises, why the ATP molecule is used and not the ADP one. There is no definite answer to this question, and we have to restrict ourselves to assumptions only. For example, the effective ATP synthesis occurs on the F_0_F_1_ synthase due to the reaction of the ATP and ADP molecules, adsorbed on the F_0_F_1_ synthase. ATP adsorption occurs with constant *K*_*d*_ ≤ 10^−12^ M, and that of ADP is less effective, *K*_*d*_ ≤ 10^−5^ M [6, p. 709]. It is assumed then that the expected value of the equilibrium constant of AMP adsorption is *K*_*d*_ ≤ 10^+2^ M. It is concluded then that the AMP adsorption and, thus, the ADP synthesis will not occur. This is a feasible reason for using ATP rather than ADP molecules in coupling.
